# Contrast-Enhanced Ultrasonography with Arrival Time Parametric Imaging as a Non-Invasive Diagnostic Tool for Liver Cirrhosis

**DOI:** 10.3390/diagnostics12123013

**Published:** 2022-12-01

**Authors:** Raluca Lupușoru, Ioan Sporea, Iulia Rațiu, Diana Lungeanu, Alina Popescu, Mirela Dănilă, Ruxandra Mare, Luciana Marc, Andrada Lascău, Tudor Voicu Moga, Felix Bende, Ana-Maria Ghiuchici, Roxana Șirli

**Affiliations:** 1 Center for Modeling Biological Systems and Data Analysis, “Victor Babes” University of Medicine and Pharmacy, 300041 Timisoara, Romania; 2 Department of Functional Sciences, “Victor Babes” University of Medicine and Pharmacy, 300041 Timisoara, Romania; 3 Department of Internal Medicine II, Division of Gastroenterology and Hepatology, “Victor Babes” University of Medicine and Pharmacy, 300041 Timisoara, Romania; 4 Advanced Regional Research Center in Gastroenterology and Hepatology, “Victor Babes” University of Medicine and Pharmacy, 300041 Timisoara, Romania; 5 Gastroenterology and Hepatology Clinic, County Emergency Hospital “Pius Brinzeu”, 300723 Timisoara, Romania; 6 Department of Internal Medicine II, Division of Nephrology, “Victor Babeș” University of Medicine and Pharmacy, 300041 Timisoara, Romania; 7 Centre for Molecular Research in Nephrology and Vascular Disease, Faculty of Medicine, “Victor Babeș” University of Medicine and Pharmacy, 300041 Timisoara, Romania; 8 Nephrology Clinic, County Emergency Hospital “Pius Brinzeu”, 300723 Timisoara, Romania; 9 Discipline of Accounting and Information System, Faculty of Economics and Business Administration, West University of Timisoara, 300115 Timisoara, Romania

**Keywords:** liver cirrhosis, CEUS, AtPI/arrival time parametric imaging, VCTE

## Abstract

Liver biopsy is the gold standard method for staging liver fibrosis, but it is an invasive procedure that is associated with some complications. There are also non-invasive techniques for assessing liver fibrosis, such as elastography and biological tests, but these techniques can fail in detection or generate false measurements depending on the subject’s condition. This study aimed to determine whether liver fibrosis can be evaluated using contrast-enhanced ultrasonography with arrival time parametric imaging using the ultrasound machine’s parametric image software, the method being called (CEUS-PAT). CEUS-PAT was performed on each subject using SonoVue as a contrast agent, and images showing liver parenchyma and the right kidney on a single screen were used for analysis in parametric imaging, which was performed using the proprietary software of the ultrasound system. The ratio between the kidney and liver arrival times was calculated. The study included 64 predominantly male (56.3%) subjects, 37 cirrhotic patients, and 27 healthy volunteers, with a mean age of 58.98 ± 8.90 years. Significant differences were found between the liver cirrhosis and healthy groups regarding CEUS-PAT, 0.83 ± 0.09 vs. 0.49 ± 0.11, *p* < 0.0001. The correlation between CEUS-PAT and VCTE was r = 0.81. The optimal cut-off value for detecting liver cirrhosis was >0.7, with an AUC of 0.98, *p* < 0.001, Se = 89.19%, Sp = 100%, PPV = 100%, and NPV = 87.1%. We demonstrate that CEUS-PAT achieves excellent performance in diagnosing liver cirrhosis and is a fast method for diagnosing liver cirrhosis that can even be applied in situations where the use of other methods is excluded.

## 1. Introduction

Cirrhosis is a chronic, diffuse, and progressive disease characterised by the development of fibrosis and the transformation of the standard liver structure into abnormal structural nodules [1]. In patients with chronic liver disease, cirrhosis and liver fibrosis stages are essential because they help determine treatment options and guide patient management, especially in the case of liver resection for primary liver malignant tumours.

Liver biopsy is the gold standard method for staging liver fibrosis, but it is an invasive procedure that is associated with some complications. There are also non-invasive techniques for evaluating liver fibrosis, such as elastography and biological tests, but these techniques may fail in detection or produce false measurement results, depending on the subject’s condition (food intake, ascites, obesity, etc.).

Although liver biopsy is considered the gold standard for assessing the severity of fibrosis and the presence of cirrhosis, the fact that only part of the liver is displayed can lead to false negative results in up to 30% of cases [2]. In addition, a biopsy is not without inherent risks and should not be repeated during follow-up [1].

Over the years, many non-invasive techniques have been used in order to assess liver fibrosis, such as biological scores and liver elastography. Liver elastography is a non-invasive technique with good accuracy in comparison with liver biopsy, the most commonly used variant being virtual controlled transient elastography (VCTE) along with point shear waves elastography (p-SWE) and two-dimensional shear waves elastography (2D-SWE). Other common elastography techniques are strain elastography (SE) and magnetic resonance elastography (MR elastography) [3]. These techniques have some limitations and confounding factors, such as elevated transaminase, fasting, pregnancy, ascites, liver congestion, obstructive jaundice, obesity, skin-to-liver distance, and the presence of pacemakers [4,5,6].

Therefore, a simple, reliable, non-invasive technique is required to evaluate liver fibrosis and cirrhosis. Studies have shown that contrast-enhanced ultrasound (CEUS) has high accuracy in diagnosing liver fibrosis [7,8]. On the other hand, in the CEUS evaluation of focal liver lesions (FLL), liver fibrosis status is important for the differentiation of lesions in normal liver and cirrhotic liver for diagnosis of hepatocarcinoma (HCC).

CEUS is a form of enhanced ultrasound imaging characterised by its safety, and it reduces the need for higher-risk, higher-cost subsequent diagnostic tests. The contrast agent does not contain dyes and does not expose patients to ionising radiation.

CEUS is based on injecting a special biocompatible ultrasound contrast agent, liquid suspensions of biocompatible gas-filled microspheres.

When injected into a patient’s cubital vein during an ultrasound examination, these microspheres flow unobstructed through the patient’s circulatory system, simulating the flow pattern of red blood cells while producing improved ultrasonic reflectivity and eventually binding to their specific targets. It has been shown that the faster the tracer reaches the liver, the more severe the fibrosis. The change in microcirculation can explain this fact. Additionally, hemodynamics in a liver with severe fibrosis are critical. [9]. The kidney was used for comparison with the liver for contrast arrival because the kidney is supplied only by arteries, and the contrast agent will travel faster. A ratio of the arrival time of the contrast agent between the right kidney and the liver was calculated. For each individual, the kidneys’ arrival time can be used as his/her own reference. If the ratio is higher, the contrast arrival time in the liver is closer to the kidney, meaning that the contrast agent came to the liver parenchyma with the arterial route explaining an imbalance in arterial-portal blood flow with arterial dominance.

This study aimed to determine whether liver fibrosis in liver cirrhosis patients can be reliably evaluated using contrast-enhanced ultrasonography with arrival time parametric imaging (CEUS-PAT).

## 2. Materials and Methods

### 2.1. Patients

We conducted a diagnostic study with a case-referent approach [10] in a tertiary gastroenterology department. Patients were enrolled during the course of one year, from January 2018 to December 2018. VCTE was the reference test.

The inclusion criteria for liver cirrhosis subjects were being older than 18 years and having a diagnosis of liver cirrhosis based on clinical, biological, endoscopic, ultrasonography, and elastography assessment; for healthy volunteers, subjects had to be older than 18 years with no evidence of any other disease, which was determined by clinical and biological parameters and by ultrasound.

Exclusion criteria were the presence of ascites, clinically significant portal hypertension, pregnancy, cardiac pacemakers, malignancy, end-stage renal diseases, heart failure, renal failure, unreliable or invalid VCTE and CAP measurements, and the elevation of aspartate aminotransferase (AST) and alanine aminotransferase (ALT) more than five times the upper limit of normal (ULN) values.

### 2.2. Ethical Consideration

All patients provided their informed consent for the procedures. The study protocol was conducted according to the Helsinki Declaration after its approval by our institution’s Ethical Committee (number 09/09.05.2016).

### 2.3. Contrast-Enhanced Ultrasonography with Arrival Time Parametric Imaging

**Figure 1:**
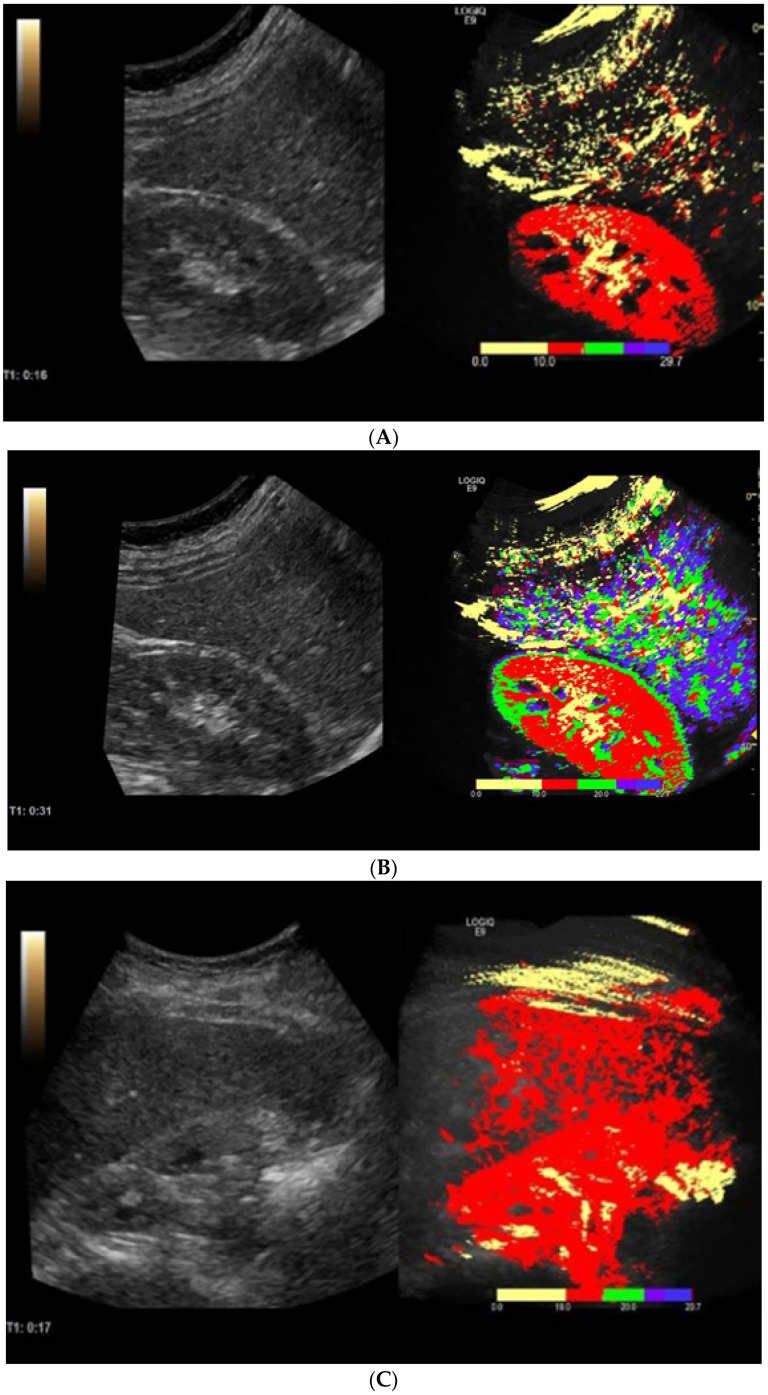

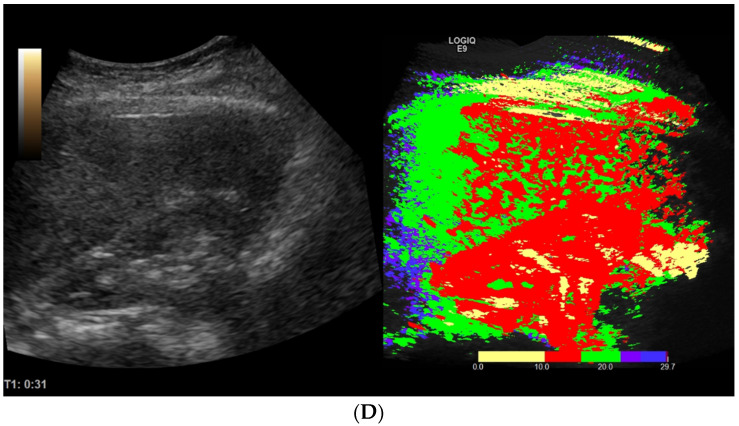
Healthy volunteer (**A**) in the first 10 s and (**B**) at the end of CEUS examination. Liver cirrhosis patient (**C**) in the first 10 s and (**D**) at the end of CEUS examination.

Ultrasonography was performed using the LOGIQ E9 (GE Healthcare, Chalfont St. Giles-UK) system, probe C1-6. CEUS was performed on each subject using SonoVue (Bracco SpA, Milan, Italy) as a contrast agent (1/2 of a vial). SonoVue is a second-generation microbubble agent (sulphur hexafluoride stabilised by a phospholipid shell). The substance was dissolved in a 5 mL saline solution. A 2.5 mL contrast substance followed by a 5 mL normal saline solution was infused in the cubital vein. Subjects were in fasting conditions for at least 12 h. Liver scanning information taken during the first 30 s following the injection of the contrast agent through the cubital vein was saved as raw data on a hard disk. The examination was performed with the patients in the left lateral position and their right arm elevated above the head, and the patients were instructed to hold their breath, especially in the first 15–20 s of the examination. Images showing liver parenchyma and the right kidney on a single screen were used for analysis, as shown in Figure 1. The examiners were blind to the patient’s medical data and elastography data.

**Figure 2:**
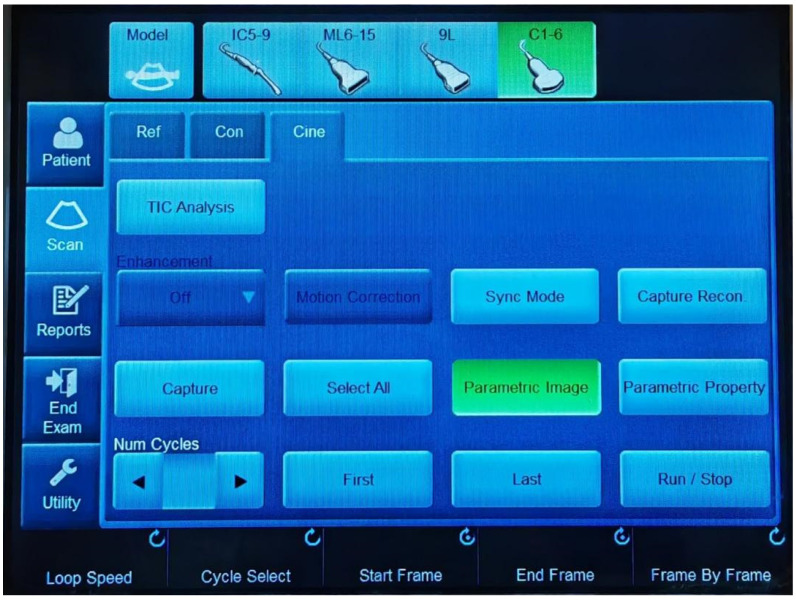
Parametric image button.

Following ultrasound acquisition, parametric imaging was performed using the dedicated image analysis software of the ultrasound system. The software “parametric image” was used to generate parametric imaging arrival time from stored video clips. This was used to visualise the contrast arrival time in colours using loops. The system would automatically prepare a colour map on the CEUS image when clicking the parametric image button (Figure 2). The colours indicate time in seconds based on a parametric colour scale of red: first 10 s, yellow: 10–15 s, green: 15–20 s, blue: 20–25 s, and purple: 25–30 s. The dedicated software provided the arrival time parametric imaging (AtPI) values of the contrast agent, the method called CEUS-PAT. The ratio between the AtPI values for the kidney and the liver was subsequently calculated. A ratio of the arrival time parametric was calculated as the ratio between the arrival time of the contrast into the kidney and the arrival time of the contrast into the liver. The software calculated the arrival time of the contrast agent [11]. The software calculated both the liver arrival time and the kidney arrival time of the contrast agent and then the ratio of these values in assessing liver fibrosis.

### 2.4. Vibration-Controlled Transient Elastography (VTCE) and Controlled Attenuation Parameter (CAP) Measurements

**Figure 3:**
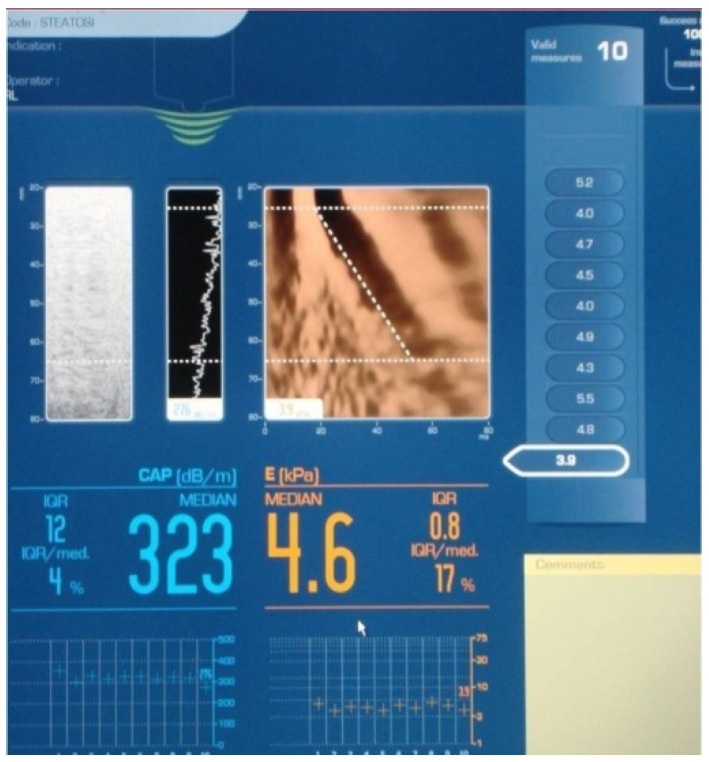
Vibration-controlled transient elastography with controlled attenuation parameter image from the software, a patient acquisition.

VCTE was performed using a FibroScan^®^ device (EchoSens, Paris, France) (Figure 3) in the right liver lobe under conditions of patient fasting for more than 4–6 h and in a supine position, with their right arm in maximum abduction, via the intercostal approach. We aimed to collect 10 valid liver stiffness measurements (LSM) for each patient. The examination was performed using the standard M probe (transducer frequency 3.5 MHz) or the XL probe (transducer frequency 2.5 MHz). M and XL probes were used according to the European recommendation for M and XL probe selection [11]. A median value of 10 valid LSM were calculated, and the results are expressed in kilopascals (kPa). Reliable measurements were defined as the median value of 10 valid LSM with an interquartile range interval/median ratio (IQR/M) < 30% [12,13]. To discriminate between the fibrosis stages, we used the following VCTE cut-off values from a metanalysis: healthy volunteers (F0-1), LS < 7 kPa, and liver cirrhosis (F = 4), LS > 12 kPa [14]. To discriminate between steatosis stages, we used the following CAP cut-off values: S1 (mild) 274 dB/m, S2 (moderate) 290 dB/m, and S3 (severe) 302 dB/m [15].

### 2.5. Surrogate Serum Fibrosis Markers

For each patient, APRI [16] and FIB-4 scores [17] were determined using the following formulas:

(1) $$\mathrm{APRI}=\frac{\frac{\mathrm{AST}\;\mathrm{Level}}{\mathrm{AST}\;\left(\mathrm{upper}\;\mathrm{limit}\;\mathrm{of}\;\mathrm{normal}\right)}}{\mathrm{Platelet}\;\mathrm{count}\;\left(10^{9}/L\right)}\times 100,$$

(2) $$\mathrm{FIB-}4=\frac{\mathrm{Age}\;\left(\mathrm{years}\right)\times \;\mathrm{AST}\;\left(U/L\right)}{\mathrm{Platelet}\;\mathrm{count}\;\left(10^{9}/L\right)\times \sqrt{ALT(U/L)}}.$$

For liver cirrhosis, the cut-off values were APRI > 2 and FIB-4 > 2.6.

### 2.6. Clinical and Biological Assessment

Anthropometric, demographic, clinical, and biological data were collected on the same day with the VCTE, CAP, and CEUS-PAT measurements. The biological data included information on haemoglobin (HB), haematocrit (HT), leucocytes (LE), thrombocytes (TR), cholesterol, triglycerides, aspartate aminotransferase (AST), alanine aminotransferase (ALT), glutamyl aminotransferase (GGT), alkaline phosphatase (AF), albumin, sodium, potassium, creatinine, urea, international normalised ratio (INR), and C-reactive protein (CRP).

### 2.7. Statistical Analysis

#### 2.7.1. Sample Size Calculation

The required number of subjects was determined based on established results [14] regarding the diagnostic accuracy of elastography for the diagnosis of cirrhosis (METAVIR F = 4) and recommendations for sample size calculation in diagnostic test studies [18]. We considered significance level alpha = 0.05, power = 0.8, sensitivity = 0.83, specificity = 0.89, and delta = 0.2 and employed an asymptotic method for estimation. The computation resulted in 31 cases and 27 controls required to guarantee design accuracy. All 64 cases enrolled from January 2018 to December 2018 were included in the data set used for assessing the accuracy of CEUS-PAT in this study.

The sample size calculation was performed using the open-source software R version 4.0.5 (*The Comprehensive R Archive Network*; https://cran.r-project.org/ (accessed on 2 August 2022)), package “MKpower” version 0.5.

#### 2.7.2. Analysis of Data

The Kolmogorov–Smirnov test was used to test the distribution of numerical variables. Qualitative variables were presented as numbers and percentages. Parametric tests (*t*-test, ANOVA) were used to assess differences between numerical variables with normal distribution; nonparametric tests (Mann–Whitney or Kruskal–Wallis tests) were used for variables with non-normal distribution. The chi-square test with Yates’ correction for continuity was used to assess the statistical significance of differences between proportions. Spearman coefficient of correlation (R) was used to evaluate the association between numerical variables. A generalised linear model (GLM) was applied to analyse the significance of clinical and paraclinical factors associated with the CEUS-PAT results; univariate and subsequent multivariate analysis was also conducted. Receiver operating characteristic (ROC) analysis was used to assess the method’s performance. Youden’s J statistic was further employed to determine the optimal cut-off value of CEUS-PAT as a diagnostic test for liver cirrhosis.

In all statistical analyses, the confidence level was 95%, with a corresponding 5% significance level. All *p*-values were two-tailed. The statistical analysis was performed using MedCalc software (v. 19.3.1, Ostend, Belgium) and IBM SPSS Statistics v. 20.0.0 (New York, NY, USA).

#### 2.7.3. Bootstrap Resampling, Sensitivity Analysis

Resampling has been acknowledged as a reliable method to determine confidence intervals for estimates derived from non-normally distributed biomedical data [19]. Bootstrapping, namely, resampling with replacement from the original sample, has been proven to lead to credible estimates for the ROC curves of diagnostic tests, especially when the sample size is small [20,21,22]. For the data set in this study, we applied the bootstrapping method to conduct a sensitivity analysis of the characteristics determined by classical asymptotic methods.

The analysis was conducted using IBM SPSS Statistics v. 20.0.0 (New York, NY, USA).

## 3. Results

### 3.1. Clinical and Biochemical Characteristics

The study included 64 subjects, 37 liver cirrhosis patients, and 27 healthy volunteers, mean age of 58.98 ± 8.90, who were predominantly of the male gender (56.3%). In the liver cirrhosis group, the majority were alcoholic cirrhosis (35.1%), followed by NAFLD (27.0%), HCV (21.6%), and HBV (16.2%) (Table 1).

Significant differences between the healthy volunteer group with the liver cirrhosis group were found upon comparison, whereby the liver cirrhosis group had lower albumin values (*p* < 0.0001) and higher CRP values (0.0007).

### 3.2. VCTE and CEUS-PAT Evaluation

**Figure 4:**
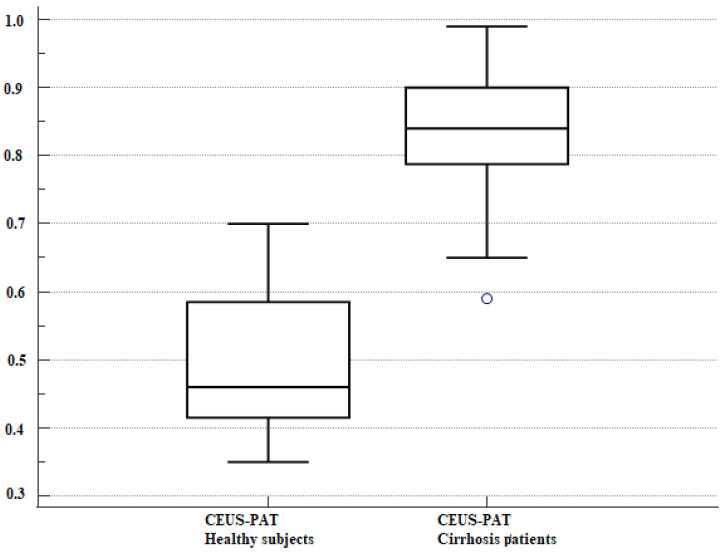
Box and whisker plot. CEUS-PAT measurements in cirrhosis patients and healthy volunteers. The boxes are proportional to the inter-quartile range (IQR) with medians marked in-between, and the whiskers are proportional to 1.5 * IQR (or trimmed to the minimum or maximum values). The bullet is an outlier.

**Figure 5:**
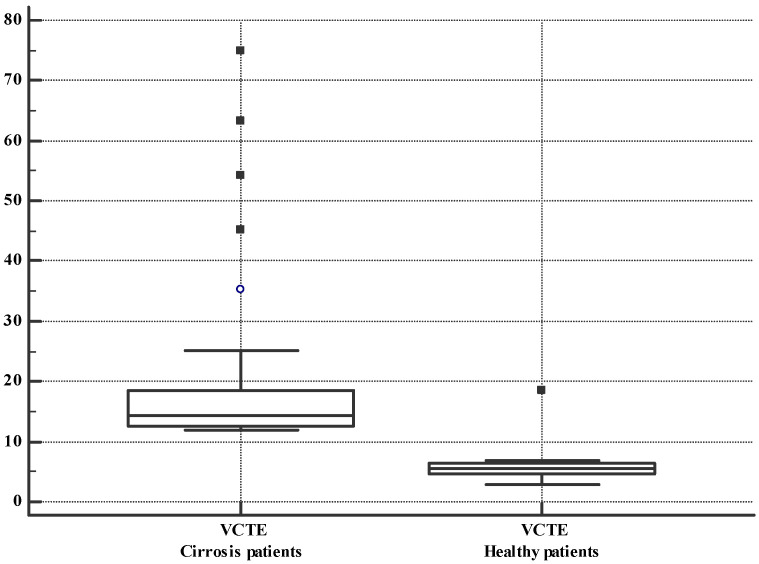
Box and whisker plot. VCTE measurements in cirrhosis patients and healthy volunteers. The boxes are proportional to the inter-quartile range (IQR) with medians marked in-between, and the whiskers are proportional to 1.5 * IQR (or trimmed to the minimum or maximum values). The bullet and the squares are outliers.

Liver cirrhosis patients had higher values of CAP, CEUS-PAT, and VCTE, as shown in Table 2 and Figure 4 and Figure 5.

### 3.3. Performance of CEUS-PAT in Detecting Liver Cirrhosis

**Figure 6:**
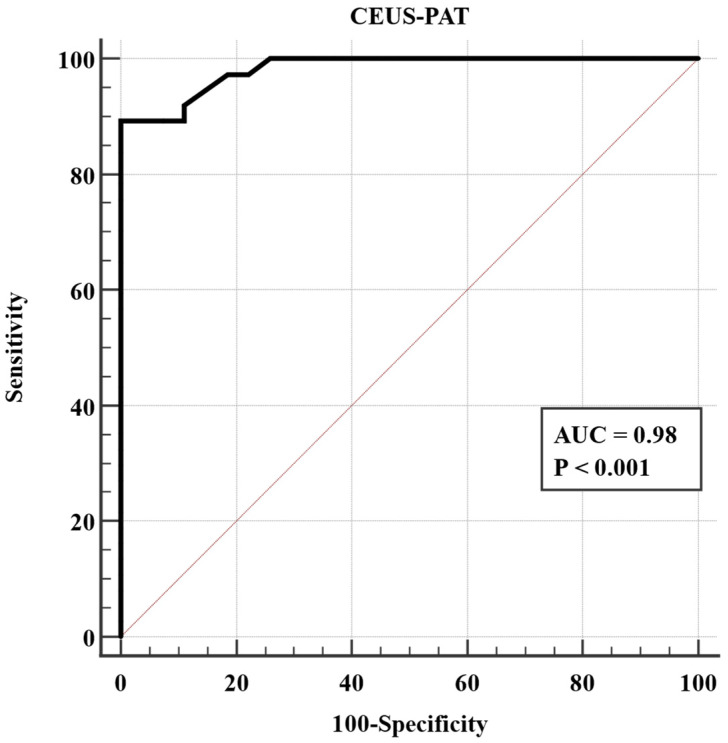
ROC analysis of CEUS-PAT in diagnosing liver cirrhosis.

Based on Youden’s index, the optimal cut-off value for detecting liver cirrhosis was a ratio of AtPI between the kidney and liver of >0.7, with an AUC of 0.98, *p* < 0.001, Se = 89.19%, Sp = 100%, PPV = 100%, and NPV = 87.1%. (Figure 6)

### 3.4. Comparison between VCTE, CEUS-PAT, FIB-4 Score, and APRI Score in Diagnosing Liver Cirrhosis

The correlation between CEUS-PAT and VCTE was strong and highly significant, with a Spearman coefficient r = 0.81 (*p* < 0.0001). Table 3 presents the two-by-two coefficients of correlation between all four indicators of cirrhosis.

**Figure 7:**
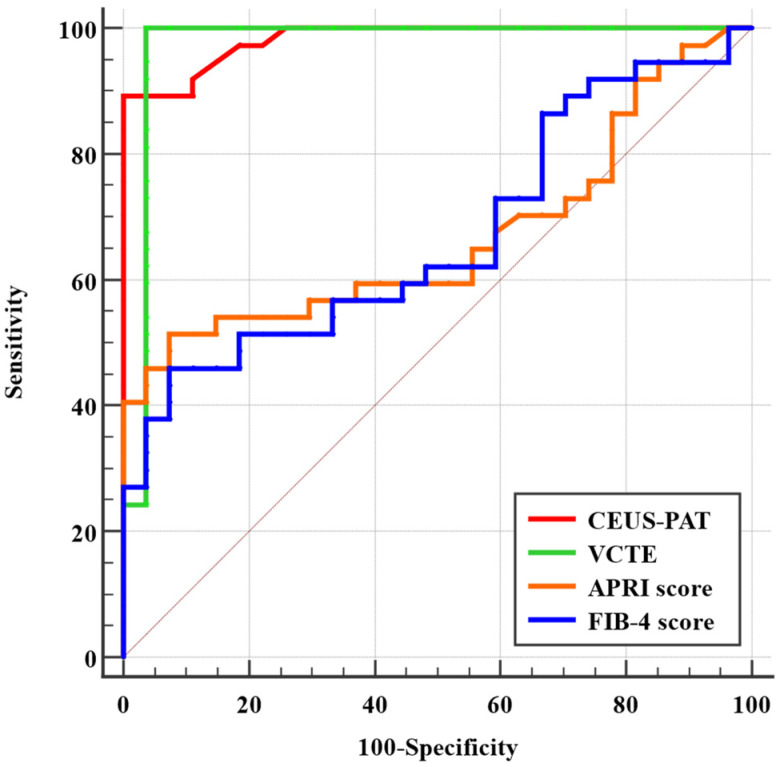
ROC curve analysis comparing the four methods.

When comparing the AUC values of the CEUS-PAT, FIB-4 score, VCTE, and APRI score in detecting liver cirrhosis, CEUS-PAT and VCTE demonstrated the best performance, with AUC = 0.98 and 0.97, followed by APRI and FIB-4 with AUC = 0.67 for both (Figure 7). 

### 3.5. Univariate and Multivariate Analysis of CEUS-PAT

To explore the significance of clinical and paraclinical factors associated with the CEUS-PAT measurements, the generalised linear model (GLM) was applied in univariate and multivariate options. In the first analysis, haemoglobin (*p* = 0.04), sodium (*p* = 0.003), AST (*p* = 0.01), ALT (*p* = 0.02), albumin (*p* < 0.0001), and severe steatosis at CAP (*p* = 0.03) were significantly associated with CEUS-PAT values. They were included in the subsequent multivariate analysis; in this latter examination, albumin was the only factor that resulted in being significantly associated with CEUS-PAT measurements. Table 4 synthesises the results.

### 3.6. Bootstrapping for Sensitivity Analysis

The bootstrap method was applied to determine the confidence interval of the differences in CEUS-PAT between the cases and controls. The confidence interval for the ROC curve of CEUS-PAT was also re-estimated. Both re-estimations led to numerical results similar to those obtained with the asymptotic methods.

## 4. Discussion

While liver elastography has gained popularity, and Fibroscan (VCTE) is considered the reference standard, certain situations do not allow us to use this approach. These situations are perihepatic ascites, obesity, pregnancy, and the presence of cardiac peacemakers. [3] Other elastography techniques have been demonstrated to perform almost as well as VCTE. However, due to the variability of external factors that could influence the results, a correlation coefficient of 1 between the methods has never been achieved, and we cannot have 100% confidence in the methods. Therefore, an alternative method, such as CEUS-PAT, is welcomed in these crucial situations. In clinical practice, while performing an abdominal ultrasound examination, we discovered liver nodules and ascites in many cases; therefore, VCTE often turns out to be inconclusive. In this setting, CEUS-PAT becomes an attractive alternative for liver stiffness measurement. On the one hand, it can confirm or rule out the diagnosis of liver cirrhosis; on the other hand, it allows for the dynamic analysis of vascularisation patterns in unclear hepatic lesions [23].

Over time, different study groups have investigated how CEUS can be applied in diagnosing liver fibrosis. This begins with the determination of the ratio of red, performed through external software. It was subsequently developed by analysing the CEUS components through the time–intensity curve (TIC) and the arrival time in the hepatic vein, artery, and portal vein [7,24].

Many previous studies have shown that CEUS can diagnose liver cirrhosis by looking at the arrival time of the contrast agent in the hepatic vein, hepatic artery, portal vein, and liver parenchyma while measuring the respective ratios of red colour [16,25].

If a patient has advanced fibrosis, their circulation will be perturbed, which is why the arrival time of the contrast agent is shortened. Another explanation could be that the contrast agent remains in the sinusoid space. Ying et al. [9] aimed to investigate the diagnosis value of CEUS for early stage liver cirrhosis in 30 patients using the same contrast agent (SonoVue) as in our study. They investigated the arrival and peak times in the hepatic artery, hepatic vein, portal vein, and liver parenchyma. The arrival time in the hepatic vein was shorter in liver cirrhosis patients, and the peak time was more prolonged than in controls. This is due to the dysfunction of Kupffer cells in liver cirrhosis. The enhancement of liver parenchyma in the late phase was darker than normal. Kim et al. [17] conducted a systematic review with a meta-analysis using CEUS to evaluate liver fibrosis, and they concluded that further studies are required.

Ridolfi et al. [26] investigated the severity of HCV, and the conclusion was that the hepatic vein arrival time decreases with fibrosis severity, a fact that is well established and in line with our study. Another study was conducted by Fujita et al. [27] on alcoholic patients, investigating the arrival time in the liver parenchyma. They compared the arrival time in the liver with that in the kidney, similar to our study, but the study was only observational. Their methods were not diagnostic; they were only comparing the arrival time of the kidney with the arrival time of the liver to see the differences. Wakui et al. [28] aimed to investigate the degree of liver progression in HCV patients using Sonazoid; cut-off values were provided for every fibrosis stage, expressed in percentages of the ratio of red and calculated using external image analysing software. Cocciolillo et al. [29] investigated CEUS performance in the diagnosis of NAFLD, measuring the blood flow of arrival time compared to transient elastography. They concluded that flow alteration precedes the development of liver fibrosis. Nasr et al. [30] conducted a study on 23 patients with NAFLD, comparing the use of biopsy to evaluate liver fibrosis. They calculated the differences in arrival time between the hepatic vein, hepatic artery, and liver parenchyma, and their results are similar to ours, finding that CEUS can be used to exclude severe cases of fibrosis.

Over the years, CEUS has also been used to investigate the presence of oesophageal varices (EV) based on time–intensity curves [31,32], and the results show that CEUS can predict EV. CEUS has also been used in animal models to investigate liver fibrosis [33,34], and it was concluded that this method has diagnostic performance for liver fibrosis, similar to our study.

Kayali et al. [35] investigated the effect of SonoVue on liver stiffness measurements. They studied 96 patients; with VCTE and SWE, measurements were taken before and after the evaluation of CEUS to see if the contrast agent affects liver stiffness, and the authors concluded that it does not. Our elastography measurements were taken before CEUS evaluation, but even when taken after, the results did not change. Recently, Yoshimine et al. [36] conducted a study on 48 patients with biliary cholangitis based on the performance of CEUS with AtPI in diagnosing liver fibrosis. They calculated the arrival time via the ratio of red with external software. The AUC values of CEUS with AiPI in diagnosing F2 and F3 were 0.77 and 0.92, similar to our AUC from CEUS-PAT of 0.98, with good performance for diagnosing liver fibrosis.

The new elastography methods that have followed VCTE demonstrate similar or even lower performance than the CEUS-PAT method in diagnosing liver cirrhosis [4]. Our study findings suggest that CEUS-PAT is an extremely accurate method for diagnosing liver cirrhosis of different aetiologies using the cut-off value of >0.7 with an AUROC value of 0.98 and very high sensibility, specificity, PPV, and NPV. On the other hand, different liver elastography methods have also proven to have good performances in predicting liver cirrhosis [37,38,39]. Still, in general, their performances are lower than CEUS-PAT.

In addition to the discriminative properties of the CEUS-PAT measurements for liver cirrhosis, we exploratorily investigated the association of the clinical and paraclinical factors with the CEUS-PAT values. Although this analysis was not intended as a regression model to identify predictors for the CEUS-PAT results, we found associations worth further exploration, such as haemoglobin, sodium, AST, ALT, albumin, and severe steatosis at CAP.

CEUS-PAT had higher values in the liver cirrhosis group compared with the healthy volunteer group, similar to VCTE values. Still, in CEUS-PAT, there was a small level of overlapping between the groups, but without any clinical revelation.

Although CEUS-PAT has been demonstrated to be a good diagnostic tool for liver fibrosis assessment over the years, the novelty of our study is that we demonstrated CEUS-PAT using a much faster method with better performance in diagnosing liver cirrhosis. The fact that we can measure the arrival time with the same ultrasound machine is an advantage, saving time for clinicians. Even if the method has a higher cost than the usual elastography methods, it saves time and money for subjects because the focal liver lesion diagnostic algorithm induces costs much lower than computer tomography and magnetic resonance imaging.

### Study Limitations

A limitation of the study is the small number of patients, calculated to assure the accuracy of this study as a proof of concept [40]. At this stage, only healthy subjects and liver cirrhosis patients were compared to validate CEUS-PAT as a reliable diagnostic method. We applied bootstrap resampling as a supplemental validation approach, and the results proved robust.

An important caveat is related to employing VCTE as a reference test in this study, which inherently limits the predicted accuracy of CEUS-PAT to the very reference we used. To compensate for this shortcoming, we analysed the correlation between the two; this analysis acted as a quantitative estimator that is symmetrical regarding the two variables and independent of the ROC area.

## 5. Conclusions

CEUS-PAT performs excellently in diagnosing liver cirrhosis and is a fast method for diagnosis of liver cirrhosis that can be applied even in situations in which the use of other methods is excluded. Future research should continue to study the different stages of liver fibrosis and strengthen the ultrasound diagnostic algorithm for focal liver lesions, particularly such that it can confirm or rule out hepatocarcinoma.

## Figures and Tables

**Table 1:** Clinical and biochemical characteristics of the study group.

Parameter	Healthy Volunteers (*n* = 27)	Liver Cirrhosis Patients (*n* = 37)	***p*-Value**
**Age (years)**	58.00 ± 8.94	59.70 ± 8.93	0.45
**Gender** **Male** **Female**	17 (62.9%) 10 (37.1%)	20 (54.0%) 17 (46.0%)	0.68
**BMI (kg/m^2^)**	25.47 ± 2.82	26.38 ± 5.29	0.37
**Haemoglobin (g/dL)**	13.46 ± 1.76	12.41 ± 1.94	0.02
**Haematocrit (%)**	37.39 ± 7.55	37.42 ± 6.94	0.98
Leucocytes (*10^9^/L)	7.96 ± 1.50	7.91 ± 2.06	0.90
**Thrombocytes (10^3^/L)**	208.77 ± 55.72	177.75 ± 97.92	0.11
**Albumin (mg/dL)**	4.05 ± 0.60	2.91 ± 0.91	<0.0001
**Natrium (mmol/L)**	138.40 ± 3.80	135.24 ± 5.16	0.006
**Potassium (mmol/L)**	4.40 ± 0.53	4.52 ± 0.57	0.38
**Urea (mg/dL)**	29.33 ± 7.08	37.43 ± 18.22	0.01
**Creatinine (mg/dL)**	0.97 ± 0.19	0.99 ± 0.28	0.80
**INR**	1.05 (0.8–1.2)	1.10 (0.8–2.3)	0.12
**AST (U/L)**	29 (13–65)	43 (17–161)	0.03
**ALT (U/L)**	35 (18–98)	45 (18–150)	0.02
**AP (U/L)**	72 (42–121)	78 (37–286)	0.23
**GGT (U/L)**	70 (16–101)	64 (14–162)	0.61
**CRP mg/L**	2.9 (1.5–123)	6.4 (1.8–128)	0.0007
**Cholesterol (mg/dL)**	150 (97–294)	157 (84–272)	0.44
**Triglycerides (mg/dL)**	126 (67–298)	113 (43–211)	0.12

ALT = alanine aminotransferase; AP = alkaline phosphatase; AST = aspartate aminotransferase; BMI = body mass index; GGT = gamma-glutamyl transferase; INR = international normalised ratio; CRP = C-reactive protein.

**Table 2:** Comparison of the CEUS-PAT and elastography measurements between the two groups.

Variable	Liver Cirrhosis Patients		Healthy Volunteers			
	*n*	Mean ± SD	*n*	Mean ± SD	Difference	*p*-Value
CAP (dB/m)	37	269.86 ± 63.46	27	223.74 ± 41.25	−46.12 (−74.07; −18.17)	0.0016
CEUS-PAT	37	0.83 ± 0.09	27	0.49 ± 0.11	−0.33 (−0.38; 0.28)	<0.0001
VCTE (kPa)	37	20.21 ± 14.97	27	5.87 ± 2.87	−14.34 (−20.19; 8.50)	<0.0001

CAP = controlled attenuated parameter; CEUS-PAT = contrast-enhanced ultrasonography with arrival time parametric imaging; CI = confident interval; *n* = total number of subjects; SD = standard deviation; VCTE = vibrating controlled transient elastography.

**Table 3:** Spearman correlation coefficients between the four methods.

		CEUS-PAT	VCTE	APRI Score	**FIB-4 Score**
CEUS-PAT	correlation coefficient	1.00	0.81	0.41	0.33
	*p*-value		0.0001	0.001	0.007
VCTE	correlation coefficient	0.81	1.0	0.38	0.21
	*p*-value	0.0001		0.001	0.08
APRI score	correlation coefficient	0.41	0.38	1.0	0.53
	*p*-value	0.001	0.001		0.0001
FIB-4 score	correlation coefficient	0.33	0.21	0.53	1.0
	*p*-value	0.007	0.08	0.0001	

**Table 4:** The results of the generalised linear model (GLM) analysis applied to the clinical and paraclinical factors associated with the CEUS-PAT measurements. Significant factors in univariate analysis were included in the multivariate model.

Parameter	Univariate Analysis			Multivariate Analysis		
	Coefficient	Std. err	*p*-Value	Coefficient	Std. err	*p*-Value
**Age (years)**	0.004	0.002	0.13			
**Gender (male)**	0.0005	0.04	0.99			
**BMI (kg/m^2^)**	0.001	0.005	0.72			
**Haemoglobin (g/dL)**	−0.02	0.01	0.04	−0.02	0.04	0.67
**Haematocrit (%)**	0.001	0.003	0.68			
Leucocytes (*109/L)	0.000007	0.01	0.99			
Thrombocytes (*103/L)	−0.0003	0.0002	0.19			
**Albumin (mg/dL)**	−0.11	0.02	<0.0001	−0.06	0.02	0.02
**Sodium (mmol/L)**	−0.1	0.004	0.003	−0.009	0.005	0.12
**Potassium (mmol/L)**	0.08	0.4	0.06			
**Urea (mg/dL)**	0.002	0.001	0.18			
**Creatinine (mg/dL)**	−0.03	0.09	0.72			
**INR**	−0.01	0.02	0.53			
**AST (U/L)**	0.001	0.006	0.01	0.0003	0.001	0.82
**ALT (U/L)**	0.001	0.006	0.02	0.0003	0.001	0.75
**AP (U/L)**	0.0009	0.0004	0.06			
**GGT (U/L)**	0.0005	0.0007	0.50			
**CRP (mg/L)**	0.0009	0.001	0.36			
**Cholesterol (mg/dL)**	−0.0002	0.0006	0.65			
**Triglycerides (mg/dL)**	−0.0005	0.0002	0.06			
**Severe steatosis at CAP**	0.007	0.001	0.03	0.002	0.001	0.23
